# Evaluation of a new very low dose imaging protocol: feasibility and impact on X-ray dose levels in electrophysiology procedures

**DOI:** 10.1093/europace/euv364

**Published:** 2015-11-20

**Authors:** Felix Bourier, Tilko Reents, Sonia Ammar-Busch, Alessandra Buiatti, Marc Kottmaier, Verena Semmler, Marta Telishevska, Amir Brkic, Christian Grebmer, Carsten Lennerz, Christof Kolb, Gabriele Hessling, Isabel Deisenhofer

**Affiliations:** Department of Electrophysiology, German Heart Center Munich, Technische Universitaet Munich, Lazarettstr. 36, München 80636, Germany

**Keywords:** Imaging, Fluoroscopy, Catheter ablation, Dose reduction, X-ray

## Abstract

**Aims:**

This study presents and evaluates the impact of a new lowest-dose fluoroscopy protocol (Siemens AG), especially designed for electrophysiology (EP) procedures, on X-ray dose levels.

**Methods and results:**

From October 2014 to March 2015, 140 patients underwent an EP study on an *Artis zee* angiography system. The standard low-dose protocol was operated at 23 nGy (fluoroscopy) and at 120 nGy (cine-loop), the new lowest-dose protocol was operated at 8 nGy (fluoroscopy) and at 36 nGy (cine-loop). Procedural data, X-ray times, and doses were analysed in 100 complex left atrial and in 40 standard EP procedures. The resulting dose–area products were 877.9 ± 624.7 µGym² (*n* = 50 complex procedures, standard low dose), 199 ± 159.6 µGym² (*n* = 50 complex procedures, lowest dose), 387.7 ± 36.0 µGym² (*n* = 20 standard procedures, standard low dose), and 90.7 ± 62.3 µGym² (*n* = 20 standard procedures, lowest dose), *P* < 0.01. In the low-dose and lowest-dose groups, procedure times were 132.6 ± 35.7 vs. 126.7 ± 34.7 min (*P* = 0.40, complex procedures) and 72.3 ± 20.9 vs. 85.2 ± 44.1 min (*P* = 0.24, standard procedures), radiofrequency (RF) times were 53.8 ± 26.1 vs. 50.4 ± 29.4 min (*P* = 0.54, complex procedures) and 10.1 ± 9.9 vs. 12.2 ± 14.7 min (*P* = 0.60, standard procedures). One complication occurred in the standard low-dose and lowest-dose groups (*P* = 1.0).

**Conclusion:**

The new lowest-dose imaging protocol reduces X-ray dose levels by 77% compared with the currently available standard low-dose protocol. From an operator standpoint, lowest X-ray dose levels create a different, reduced image quality. The new image quality did not significantly affect procedure or RF times and did not result in higher complication rates. Regarding radiological protection, operating at lowest-dose settings should become standard in EP procedures.

**What's new?**

- By now, the currently available low-dose X-ray imaging protocol is operated at 23 nGy (fluoroscopy) and at 120 nGy (cine-loop).
- Siemens AG has recently developed a new very low dose protocol especially designed for EP procedures, which is operated at 8 nGy (fluoroscopy) and at 36 nGy (cine-loop).
- This study demonstrates that working at these lowest-dose levels is feasible and safe, the reported dose–area products are very low when compared with previously published X-ray dose data.

## Introduction

Fluoroscopy is the basic imaging modality in the electrophysiology (EP) catheterization laboratory. For complex EP interventions, in particular atrial fibrillation ablation procedures, 3D navigation systems, and preprocedural 3D imaging are commonly employed to supplement fluoroscopic imaging. Previous studies showed that the use of 3D navigation systems significantly decreases radiation exposure in EP procedures.^1–4^

Adaptive use of X-ray collimation and principles of radiological protection should generally be applied to keep radiation exposure as low as possible.^5^ Since fluoroscopy systems are technically optimized for high-resolution angiography, there is room for dose reduction when used for EP interventions not requiring detailed resolution. By now, the lowest available clinical acquisition settings for fluoroscopic EP procedures on an *Artis zee* angiography system (Siemens AG, Forchheim, Germany) are operated at 23 nGy detector entrance dose per fluoroscopy pulse. In this study, we present and evaluate a new very low dose fluoroscopy protocol (Siemens AG), which was especially designed for EP interventions, offering fluoroscopy operated at 8 nGy detector entrance dose per fluoroscopy pulse.

## Methods

### Study overview

**Figure 1:**
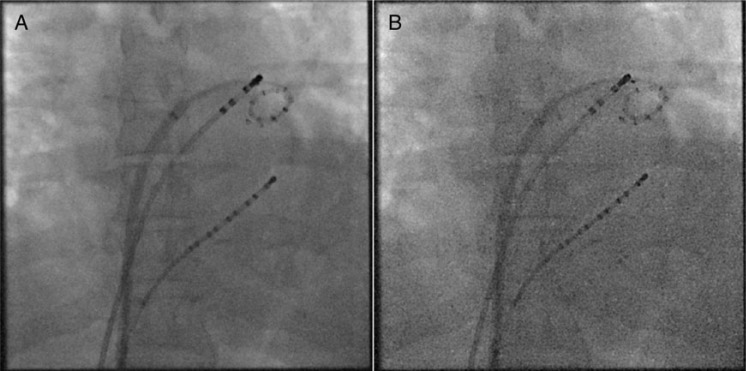
Anterior-posterior view fluoroscopic acquisitions using the old low-dose protocol (*A*) and the new lowest-dose protocol (*B*). The images show coronary sinus, circular mapping, and RF-ablation catheters.

From October 2014 to March 2015, overall 140 consecutive patients underwent an EP study on an *Artis zee* angiography system. All patients gave written informed consent before the procedure. The study was approved by the ethics committee. From October 2014 to December 2014, fluoroscopy was operated at the standard available low-dose EP imaging protocol. From January 2015 to March 2015, fluoroscopy was operated at a new very low dose protocol. For each EP study, procedural data were documented and stored for later analysis. *Figure 1* shows an example of a fluoroscopic image using the old standard low dose (*A*) and the new lowest dose (*B*) acquisition protocol.

### Fluoroscopy protocols

The detector entrance dose settings of the available standard low-dose and the new lowest-dose protocols are summarized in *Table 1*. In both protocols, cine-loops were acquired with 7.5 fps using a tube currency of 81 kV. Fluoroscopic images were acquired with 3 fps using a tube currency of 90 kV.

**Table 1:** Detector entrance dose settings for low-dose and lowest-dose protocol

Detector entrance dose	Low dose (nGy)	Very low dose (nGy)
Cine-loop acquisition	120	36
Fluoroscopy acquisition	23	8

### Ablation procedures

All EP procedures included in this study were carried out in the same laboratory by four experienced operators. For analysis, patient details were documented and the procedures were divided into complex left atrial procedures (atrial fibrillation- and atypical flutter-ablation) and standard procedures (typical flutter-, AVNRT-, WPW-, and PVC-ablation). The procedures were performed according to the current clinical standards. All left atrial and PVC-ablation procedures were supported by CARTO 3 (Biosense Webster, Diamond Bar, CA, USA) or EnSite Velocity (St Jude Medical, St Paul, MN, USA).

### Procedural data

For all procedures, X-ray times, dose–area products (DAPs; µGym²), and Air kerma (AK; mGy) values were documented. The AK represents absorbed radiation dose and correlates with deterministic radiation effects. The DAP is defined as absorbed dose multiplied by the irradiated area and it correlates with stochastic radiation effects. For left atrial procedures, procedural times for each step (puncture, mapping, and ablation) as well as cine-loop times, angulations and doses were documented separately. Procedure and radiofrequency (RF) times and complications were documented as further study parameters.

### Clinical outcome

A 6-month follow-up assessed the clinical outcome of the ablation procedures. For atrial fibrillation and atrial flutter, any asymptomatic or symptomatic episode lasting >30 s in a routinely performed 7-day long-term electrocardiogram (ECG) or symptomatic arrhythmic episodes lasting >30 s without ECG documentation were counted as a recurrence. In AVNRT, WPW, and PVC patients, symptomatic arrhythmic episodes or ECG documentations of the clinical arrhythmia were counted as a recurrence.

### Statistics

The mean values were calculated as the arithmetic average ± standard deviation. Comparisons between groups were made by *χ*^2^ test or Fisher's exact test, for categorical variables and unpaired *t*-test or one-way analysis of variance for normally distributed variables. A *P*-value of <0.05 was considered statistically significant. Cine-loop doses were calculated per cine-loop frame and fluoroscopy dose was calculated per fluoroscopy time.

## Results

Overall 100 left atrial and 40 standard procedures were analysed, including 237 cine-loops. All operators were able to carry out the procedures using the very low X-ray dose protocol. Switching to higher dose levels was not necessary in any case. Relevant patient characteristics and treated arrhythmias are summarized in *Tables 2* and *3*.

**Table 2:** Patient details of both groups

Patient details (*n* = 140)	Low-dose group	Lowest-dose group	*P*-Value
Age (years)	57.3 ± 15.3	58.8 ± 16.2	0.57
Female sex (%)	35%	32%	0.71
BMI (kg/m²)	26.2 ± 2.9	25.9 ± 3.6	0.58
Body surface area (m²)	2.01 ± 0.17	1.98 ± 0.24	0.56
Ejection fraction (%)	55.2 ± 9.3%	54.5 ± 10.4%	0.55
Hypertension, *n* (%)	74 (53%)	83 (59%)	0.28
Diabetes mellitus, *n* (%)	15 (11%)	11 (8%)	0.41
Coronary artery disease, *n* (%)	17 (12%)	25 (18%)	0.18

**Table 3:** Numbers of treated arrhythmias in both groups

Treated arrhythmia (*n* = 140)	Low-dose group	Lowest-dose group
Atrial fibrillation, *n*	35	32
Atypical flutter, *n*	15	18
Typical flutter, *n*	5	3
Accessory pathway, *n*	3	3
AVNRT, *n*	6	8
PVC, *n*	6	6

The procedural and clinical outcome data are shown separately for left atrial and for standard procedures (*Tables 4* and *5*). Both reported complications were arteriovenous fistulas.

**Table 4:** Procedural and clinical data of left atrial procedures

Complex procedures (*n* = 100)	Low-dose group	Lowest-dose group	*P*-Value
Procedure time (min)	132.6 ± 35.7	126.7 ± 34.7	0.40
Puncture time (min)	12.9 ± 5.2	11.3 ± 6.7	0.18
Mapping time (min)	11.7 ± 4.3	10.6 ± 4.8	0.23
RF time (min)	53.8 ± 26.1	50.4 ± 29.4	0.54
Complications, *n*	1	1	1.0
X-ray time (min)	10.1 ± 7.6	8.6 ± 6.2	0.28
Six-month follow-up success (%)	70%	72%	0.82

**Table 5:** Procedural and clinical data of standard procedures

Standard procedures (*n* = 40)	Low-dose group	Lowest-dose group	*P*-Value
Procedure time (min)	72.3 ± 20.9	85.2 ± 44.1	0.24
RF time (min)	10.1 ± 9.9	12.2 ± 14.7	0.60
Complications, *n*	0	0	1.0
X-ray time (min)	7.1 ± 5.4	8.7 ± 6.4	0.40
Six-month follow-up success (%)	95%	95%	1.0

**Figure 2:**
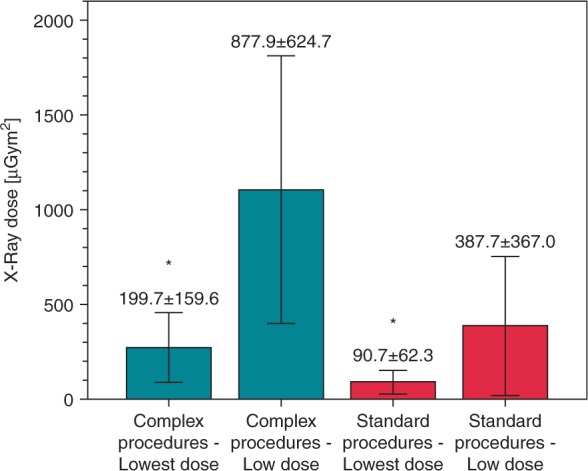
X-ray DAPs resulting from standard low-dose and new lowest-dose settings in standard and complex left atrial procedures. The reduction of DAPs was highly significant (*P* < 0.01).

The use of the very low dose protocol facilitated a mean X-ray dose reduction of 77.3% for complex and of 76.6% for standard procedures. *Figure 2* shows the overall resulting X-ray DAPs. *Table 6* summarizes AK values, DAPs per frame regarding different C-arm angulations, and DAPs per fluoroscopy time.

**Table 6:** Air kerma values, cine-loop DAPs/frame for different angulations, and DAP per fluoroscopy time

Parameter	Low-dose group	Lowest-dose group	*P*-Value
Cumulative AK standard (mGy)	65.4 ± 75.5	15.6 ± 19.8	<0.01
Cumulative AK complex (mGy)	110.7 ± 67.6	24.6 ± 17.68	<0.01
Cine-loop DAP (µGym²)	170.2 ± 179.0	50.1 ± 37.8	<0.01
Cine-loop DAP/frame LAO (µGym²)	4.5 ± 6.3	1.0 ± 0.7	<0.01
Cine-loop DAP/frame anterior-posterior (µGym²)	1.3 ± 0.7	0.4 ± 0.4	<0.01
Cine-loop DAP/frame RAO (µGym²)	2.6 ± 0.8	0.74 ± 0.7	<0.01
Fluoroscopy DAP/s (µGym²/s)	1.73 ± 1.21	0.42 ± 0.24	<0.01

## Limitations

The study was based on retrospective analysis of procedural data. Owing to national radiological protection law, it was not possible to design the study as a randomized controlled trial. Basically, X-ray dose levels are affected by angulation of the angiography system and collimation. The documentation of angulation was only available for cine-loops. However, we expected that the operators applied collimation and angulation in their same used manner when working at low-dose and lowest-dose fluoroscopy.

## Discussion

In this study, we present a new very low dose imaging protocol which was especially designed for EP procedures. To our knowledge, it is the first evaluation of these acquisition settings for EP procedures. The resulting dose levels of 199.7 ± 159.6 µGym² (DAP) and 24.6 ± 17.68 mGy (AK) for left atrial ablation procedures are very low when compared with previously published DAP levels between 789 and 11 199 µGym².^6–10^ The AK and DAP data show that lowest-dose fluoroscopy can significantly extenuate both the deterministic (e.g. skin injuries) as well as stochastic (e.g. risk of malignancy) effects of radiation exposure.

In the low-dose and lowest-dose acquisition protocols, the X-ray tube voltage was identically chosen (81 kV for cine-loops and 90 kV for fluoroscopy) which prevented that softer X-ray radiation might create better image quality in one group but be more harmful to tissue.

In the past years, the increased use of 3D navigation systems and operators' awareness for radiological protection led to a reduction of X-ray exposure in EP interventions, especially in complex procedures.^1–3^ Recent publications demonstrated that completely non-fluoroscopic ablation procedures based on 3D systems and intracardiac echocardiography are feasible.^11–15^ However, current real-world data, as recently presented in a European Heart Rhythm Association survey by Estner *et al*.,^16^ show that fluoroscopy still remains the basic imaging modality in EP procedures. For complex procedures, a median fluoroscopy time of 16 min and a cumulative DAP of 6100 µGym² are reported. As long as fluoroscopy remains a commonly applied imaging technology, the implementation of lowest-dose fluoroscopy protocols to achieve a significant reduction of radiation exposure remains highly preferable.

Nof *et al*.^17^ recently published the successful implementation of a custom made very low dose fluoroscopy protocol allowing a significant reduction of radiation doses. In their study, the correlation of patient body mass index (BMI) and system angulation on radiation exposure was evaluated, which showed an exponential increase of radiation dose depending on patient BMI and left anterior oblique (LAO) angulation.

Previous paediatric studies dealing with strategies of dose reduction in EP procedures showed that the use of radiation safety protocols, lowered detector entrance doses, and individual adaption of fluoroscopy frame rates leads to a significant reduction in X-ray dose exposure without increasing procedure times, complication rates, or outcome.^18–23^ Also the removal of the scatter grid, which resulted in poorer image quality, has been shown to reduce radiation by as much as two-fold.^24^

From an operator standpoint, lowest X-ray dose levels create a different, reduced image quality (*Figure 1*). However, although unfamiliar, the new image quality did not significantly affect procedure times, clinical outcome, or RF times and did not result in higher complication rates.

## Conclusion

The data demonstrate that applying lowest dose imaging protocols especially designed for EP procedures reduce X-ray dose levels by 77% compared with the current standard low-dose protocol. Regarding radiological protection, operating at lowest-dose settings should become standard in EP procedures.

**Conflict of interest:** none declared.
